# Evaluation of short video quality and reliability of non-invasive prenatal testing on TikTok and Bilibili platforms: a cross-sectional study

**DOI:** 10.1186/s12884-026-08965-x

**Published:** 2026-03-20

**Authors:** Suxiao Liu, Yiqi Zhao, Zixing Zhong, Jiamin Luo

**Affiliations:** 1 Center for Reproductive Medicine, Department of Obstetrics, Zhejiang Provincial People’s Hospital (Affiliated People’s Hospital, Hangzhou Medical College), Hangzhou, Zhejiang China; 2 Center for Reproductive Medicine, Department of Ultrasound Medicine, Zhejiang Provincial People’s Hospital (Affiliated People’s Hospital, Hangzhou Medical College), No. 158 Shangtang Road, Hangzhou, Zhejiang 310014 China

**Keywords:** Non-Invasive Prenatal Testing (NIPT), Short video, TikTok, Bilibili, Social media

## Abstract

**Background:**

Non-invasive prenatal testing (NIPT) is a genetic test to screen fetal chromosomal abnormalities in pregnancy. Public reliance on short-video platforms for health information in China is increasing, yet the quality of such online content remains unassessed. This study aimed to evaluate the quality and reliability of videos related to NIPT on major Chinese social media platforms.

**Methods:**

A cross-sectional study was conducted to analyze short videos related to NIPT on TikTok and Bilibili. Data of video duration, video source and user interaction metrics were collected for statistical analysis. Video quality assessment tools, such as the Global Quality Score (GQS), modified DISCERN, Journal of the American Medical Association (JAMA) benchmark criteria and the Video Information and Quality Index (VIQI) were used for evaluation and comparison. Statistical analyses included descriptive analysis, non-parametric tests and the Spearman correlation analysis.

**Results:**

A total of 180 videos (80 from Bilibili, 100 from TikTok) were analyzed. Bilibili videos were significantly longer and scored higher in median GQS (3.00 vs. 2.50), mDISCERN (3.00 vs. 2.00), and VIQI (12.00 vs. 8.00) compared to TikTok (all *P* < 0.05), though JAMA benchmark criteria were similar. TikTok videos generated substantially higher user engagement (likes, collections, shares, comments). Videos uploaded by medical professionals received higher engagement and quality scores than those from institutions or individual users. Correlation analyses revealed weak or inconsistent associations between user engagement metrics and video quality scores on both platforms, indicating that popular content was not reliably accurate.

**Conclusion:**

The quality and reliability of short videos about NIPT on these platforms need to be improved. Platform regulators should also strengthen the supervision of the quality of short videos.

**Supplementary Information:**

The online version contains supplementary material available at 10.1186/s12884-026-08965-x.

## Introduction

Non-invasive prenatal testing (NIPT) has emerged as a transformative tool in prenatal care, enabling the screening for common fetal chromosomal aneuploidies [1]. Since its clinical introduction, NIPT has been widely adopted given its high sensitivity and specificity. It offers a safe and reliable alternative to traditional invasive diagnostic procedures in aneuploidies, such as trisomy 21 [2]. Its growing integration into routine prenatal screening protocols globally has made it a frequent topic of discussion among expectant parents.

Currently, social media platforms, particularly short-video applications like TikTok and Bilibili, have become a dominant source for health information. TikTok is a globally well-known platform, and its Chinese version is also known as Douyin [3]. Bilibili is a popular Chinese social media platform, similar to Youtube [4]. These platforms leverage engaging, algorithm-driven content to reach broad and diverse audiences. However, the unregulated and user-generated nature of this content raises significant concerns regarding quality, accuracy, and reliability [5, 6]. Simplifying complex medical topics for brevity and engagement can perpetuate misinformation and foster unrealistic expectations, thereby potentially impacting viewers’ health behaviors and decision-making.

Previous studies have evaluated social media content related to reproductive health topics, including gestational diabetes and uterine fibroids. However, NIPT-related information on these platforms has not yet been systematically examined [5, 7]. NIPT is distinct from these topics as it involves genetic testing with potentially further decisions about pregnancy continuation. Its unique psychological implications for expectant parents make accurate information dissemination particularly critical. Given the widespread and active discussions surrounding NIPT online, a comprehensive assessment of its content quality and reliability is both timely and warranted. Therefore, this cross-sectional study aimed to evaluate the informational quality and reliability of short videos concerning non-invasive prenatal testing on two major Chinese social media platforms: Douyin (Chinese TikTok) and Bilibili. This research seeks to characterize the landscape of NIPT information online and provide evidence-based insights to guide healthcare professionals, policymakers, and users in critically navigating this digital health information environment.

## Materials and methods

### Data collection and screening

In this cross-sectional study, we conducted a video search using the Chinese translation of “non-invasive prenatal testing (NIPT)”, “无创DNA” as keyword on Bilibili (www.bilibili.com) and Douyin (Chinese TikTok: www.douyin.com). Then we screened top 140 videos from each platform based on default sorting algorithm to simulate the experience of a typical user. Data screening was conducted on a single day, 4th December, 2025. To minimize biases that may be introduced by personalized recommendation features, researchers deliberately created new user accounts in incognito browsing mode on each platform for data screening and collection. The inclusion criteria were NIPT relevant videos in Chinese with duration more than 10 s. The exclusion criteria were: irrelevant, or duplicate videos, or videos with promotional advertisements [8]. For duplicate videos, the earliest video was retained for analysis. Then, basic information of the video, including the title, identity of uploaders, the duration of the video, and the social engagement data, such as likes, comments, sharing and collection were extracted [9]. Videos were divided into three groups according to their source: 1) Professionals (healthcare professionals including obstetricians, gynecologists and other doctors or nurses; 2) Institutions (defined as medical or educational organisations’ accounts); 3) Individuals (defined as general users or laypersons).

### Video quality and reliability assessment

The quality and reliability of information of the videos were evaluated by Global Quality Scale (GQS), Modified Decision-making Information Support Criteria for Evaluating the Reliability of Non-randomised Studies (mDISCERN), Journal of American Medical Association (JAMA) benchmark criteria, and Video Information and Quality Index (VIQI). The GQS was rated from 1 to 5; Higher scores indicate higher quality [10]. The mDISCERN evaluates five items: (1) clarity of aims, (2) use of reliable source, (3) balance of information, (4) additional information, (5) area of uncertainty. Each score 0 or 1, higher scores indicate better reliability [11]. The JAMA transparency score was also adopted for evaluation. It contains 4 yes/no items, each “yes” scores 1 point, while “no” scores 0 point. The total score ranges from 0 to 4 points [12]. VIQI evaluates video quality across four domains: information flow, clarity, quality, and consistency. Each domain is scored on a 5-point Likert scale, yielding a total score ranging from 0 to 20, with higher scores indicating better quality [13]. All assessments were reviewed independently by two raters (Yiqi Zhao and Suxiao Liu), both of whom are physicians with clinical experience on prenatal diagnosis. Any disagreement in rating was resolved by discussion or a third investigator (Jiamin Luo). Cohen’s coefficients were used to quantify the agreement between two raters (Supplementary Table 1). κ > 0.8 indicates excellent agreement, values between 0.6 and 0.8 are considered significant, values between 0.4 and 0.6 are considered moderate, and values < 0.4 are considered poor.

### Ethical considerations

The research materials in this study all came from the video content publicly available on TikTok and Bilibili platforms. No clinical data, human samples or experimental animal data were collected, and no interactions with users were involved. This study was waived from the institutional ethical review process. The large language model (LLM) DeepSeek was used solely for language editing, and there is no AI-generative content in this manuscript.

### Statistical analysis

Analyses were performed with the use of SPSS, version 27.0 (IBM). Descriptive statistics are presented as median (IQR) given the non-parametric distribution of the data. The Mann-Whitney U test was used to compare quality differences across platforms (TikTok vs. Bilibili). The Kruskal-Wallis test was used to assess video quality across different groups of uploaders. Spearman rank correlation coefficients were calculated to assess the association between quality ratings and interaction metrics. Cohen’s κ coefficients were used to assess inter-rater reliability between raters. Statistical significance was set at *P* < 0.05 for all tests.

## Result

### Video selection process

**Fig. 1:**
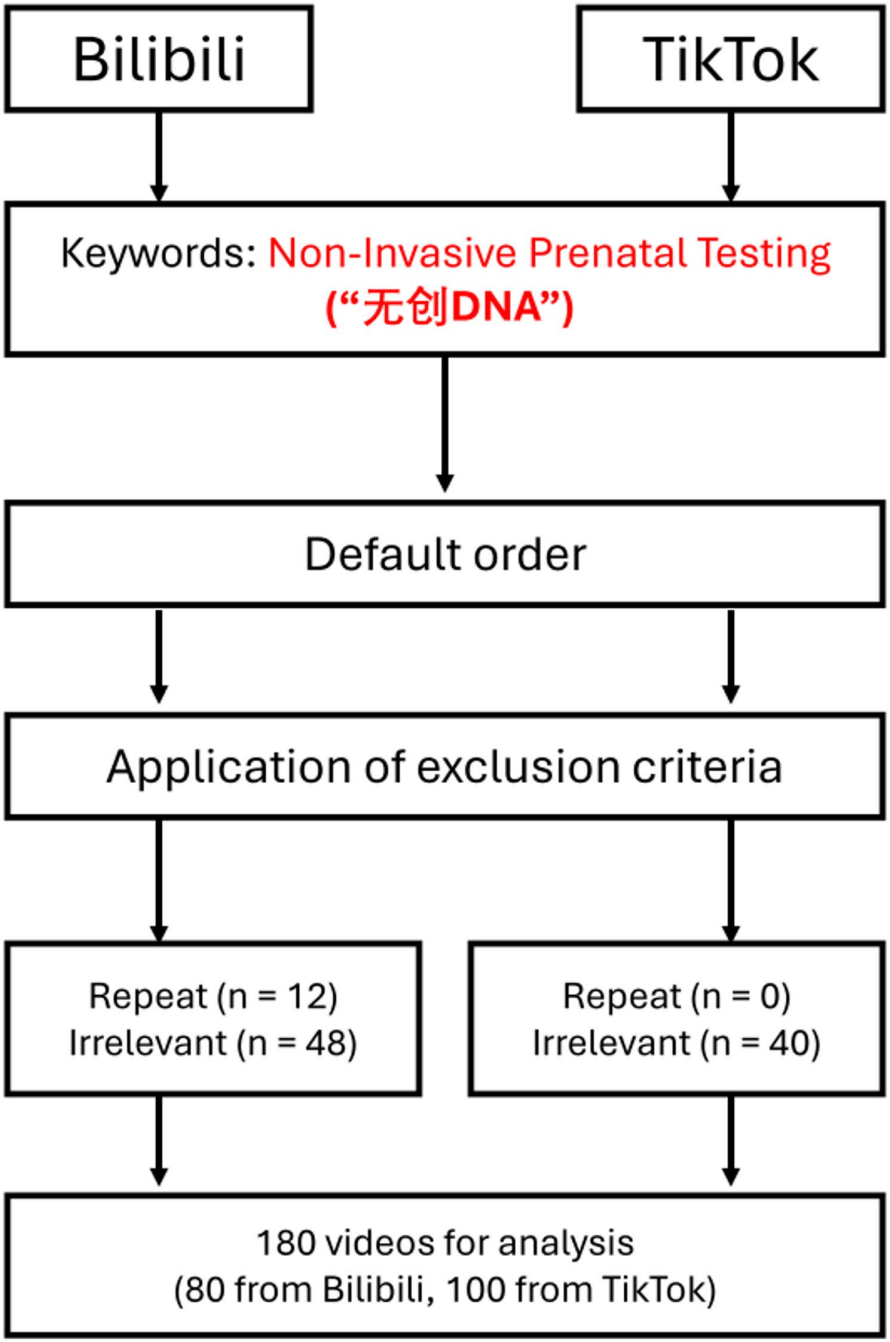
Search strategy and video selection process

According to the default search results of the video platform, the first 140 videos were obtained on TikTok and Bilibili, respectively with the keywords “non-invasive prenatal testing” in Chinese. By applying the exclusion criteria, repeated and irrelevant videos were excluded, and 180 valid videos were finally included for further statistical analysis. 80 videos were from Bilibili, while 100 from TikTok. The detailed search strategy and video selection process are shown in Fig. 1.

### Video characteristics

The basic characteristics of the included videos from both platforms are presented in Table 1. In terms of video duration, the median video duration of videos from Bilibili was 96.00 (66.00, 183.00), which was significantly longer than 64.00 (43.75, 96.00) of TikTok (*P* < 0.001). By contrast, videos from TikTok showed substantially higher user engagement. The median numbers of social engagement metrics, such as likes, collections, comments and shares were 933.00 (253.75, 2194.75), 299.00 (61.00, 878.00), 379.00 (121.75, 1128.25) and 740.50 (111.75, 2194.75), respectively, which were significantly higher than videos on Bilibili (*P* < 0.01 for all).

**Table 1:** Basic characteristics of videos from both platforms

Variables	Bilibili (*n* = 80)	TikTok (*n* = 100)	Effect size	*P*-value
Video length, M (Q₁, Q₃)	96.00 (66.00, 183.00)	64.00 (43.75, 96.00)	Z = -4.77	< 0.001
Likes, M (Q₁, Q₃)	9.00 (3.00, 24.50)	933.00 (253.75, 2194.75)	Z = -9.91	< 0.001
Collections, M (Q₁, Q₃)	3.00 (1.00, 13.25)	299.00 (61.00, 878.00)	Z = -9.22	< 0.001
Comments, M (Q₁, Q₃)	1.00 (0.00, 5.00)	379.00 (121.75, 1128.25)	Z = -10.37	< 0.001
Shares, M (Q₁, Q₃)	4.00 (1.00, 16.00)	740.50 (111.75, 1964.00)	Z = -9.80	< 0.001
GQS, M (Q₁, Q₃)	3.00 (3.00, 4.00)	3.00 (2.00, 3.00)	Z = -2.31	< 0.05
mDISCERN, M (Q₁, Q₃)	3.00 (2.00, 3.00)	2.00 (2.00, 3.00)	Z = -2.59	< 0.05
JAMA, M (Q₁, Q₃)	2.00 (1.00, 2.00)	2.00 (1.00, 2.00)	Z = -1.67	0.095
VIQI, M (Q₁, Q₃)	12.00 (8.00, 15.25)	8.00 (5.00, 14.00)	Z = -3.72	< 0.001
Uploader, *n* (%)			χ^2^ = 16.38	< 0.001
Individuals	39 (48.75)	36 (36.00)		
Institutions	22 (27.50)	12 (12.00)		
Professionals	19 (23.75)	52 (52.00)		

*M* median, *Q*_1_ 1^st^ quartile, *Q*_3_ 3^rd^ quartile, *Z *Mann-Whitney test, *χ*^2^ Chi-square test

**Fig. 2:**
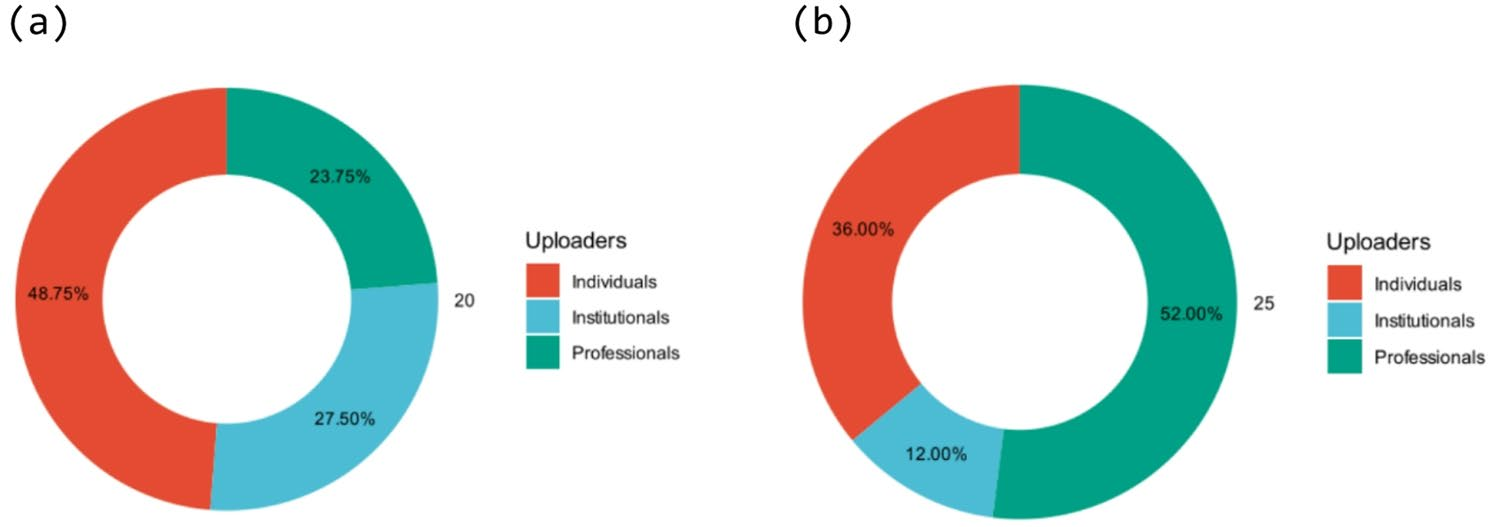
Distribution of NIPT-related videos by uploader type across both platforms: (**a**) Bilibili, (**b**) TikTok

The distribution of uploader types also varied significantly (χ^2^ = 16.38, *p* < 0.001). Bilibili was dominated by individuals (48.75%) and institutions (27.50%), while TikTok was overwhelmingly populated by content from professionals (52.00%), followed by institutionals (36.00%) (Table 1; Fig. 2).

Among uploaders, the median duration of videos uploaded by experts was 65.00 (43.50,96.00), which was significantly shorter than that of other types of uploaders (*P* = 0.016), but the social engagement metrics were more prominent. Healthcare professionals had significantly higher number of social engagement metrics (median number of likes, collections, comments and shares: 916.00 (139.50,2844.00), 276.00 (44.50,903.00), 290.00 (44.50,1211.00) and 675.00 (49.00,2065.50), respectively) than those of institutional and individual users (all *P* < 0.001; Table 2).

**Table 2:** Video characteristics of different uploaders

Variables	Individuals (*n* = 75)	Institutions (*n* = 34)	Professionals (*n* = 71)	Effect size	*P*-value
Video length, M (Q₁, Q₃)	91.00 (55.00, 176.00)	86.00 (61.75, 113.25)	65.00 (43.50, 96.00)	χ^2^ = 8.30	< 0.05
Likes, M (Q₁, Q₃)	23.00 (7.50, 362.00)	28.00 (5.25, 454.25)	903.00 (74.50, 2528.00)	χ^2^ = 27.98	< 0.001
Collections, M (Q₁, Q₃)	12.00 (3.00, 104.00)	18.50 (1.00, 138.25)	208.00 (20.50, 850.50)	χ ^2^ = 22.44	< 0.001
Comments, M (Q₁, Q₃)	8.00 (1.00, 218.50)	5.00 (0.00, 124.50)	253.00 (8.00, 1155.50)	χ ^2^ = 20.95	< 0.001
Shares, M (Q₁, Q₃)	16.00 (2.00, 218.50)	16.00 (4.00, 367.00)	380.00 (26.50, 2065.50)	χ ^2^ = 22.22	< 0.001
GQS, M (Q₁, Q₃)	3.00 (2.00, 3.00)	3.00 (3.00, 3.75)	3.00 (3.00, 4.00)	χ ^2^ = 13.38	< 0.001
mDISCERN, M (Q₁, Q₃)	2.00 (2.00, 3.00)	3.00 (2.00, 3.00)	2.00 (2.00, 3.00)	χ ^2^ = 1.72	0.423
JAMA, M (Q₁, Q₃)	2.00 (1.00, 2.00)	2.00 (2.00, 2.00)	2.00 (1.00, 2.00)	χ ^2^ = 4.35	0.114
VIQI, M (Q₁, Q₃)	8.00 (5.00, 13.50)	12.00 (8.25, 15.50)	10.00 (6.00, 14.00)	χ ^2^ = 6.27	< 0.05

*M *median, *Q*_1_ 1^st^ quartile, *Q*_3_ 3^rd^ quartile. *χ*^2^ Kruskal-wallis test

### Video content and quality

**Fig. 3:**
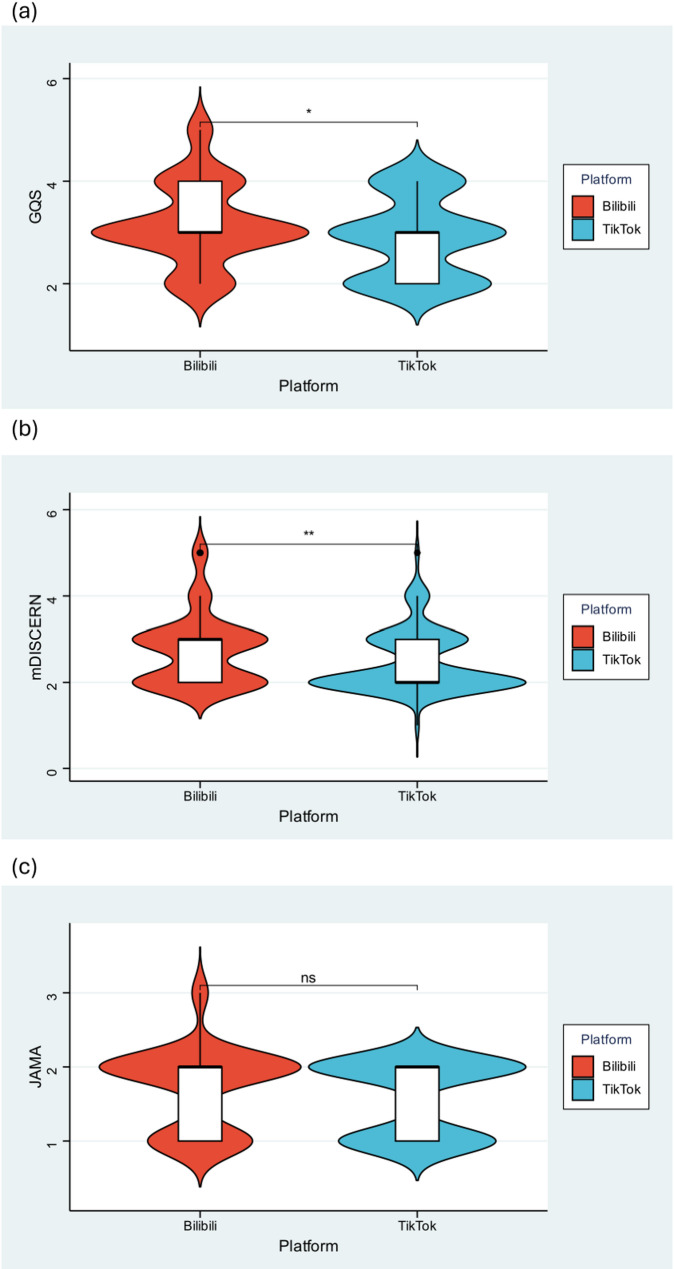
GQS, mDISCERN, JAMA scores and the quality distribution of videos related to NIPT across both platforms: (**a**) GQS, (**b**) mDISCERN, (**c**) JAMA

Regarding video quality compared between the two platforms, the median GQS, modified DISCERN, JAMA benchmark criteria, and VIQI of Bilibili videos were 3.00 (3.00, 4.00), 3.00 (2.00, 3.00), 2.00 (1.00, 2.00), and 12.00 (8.00, 15.25), respectively. The scores of GQS and mDISCERN were generally concentrated between 2 and 4 on both platforms (Fig. 3). The median scores were slightly lower in TikTok videos, except the JAMA benchmark, which was comparable between the two platforms (Table 1; Fig. 3).

**Fig. 4:**
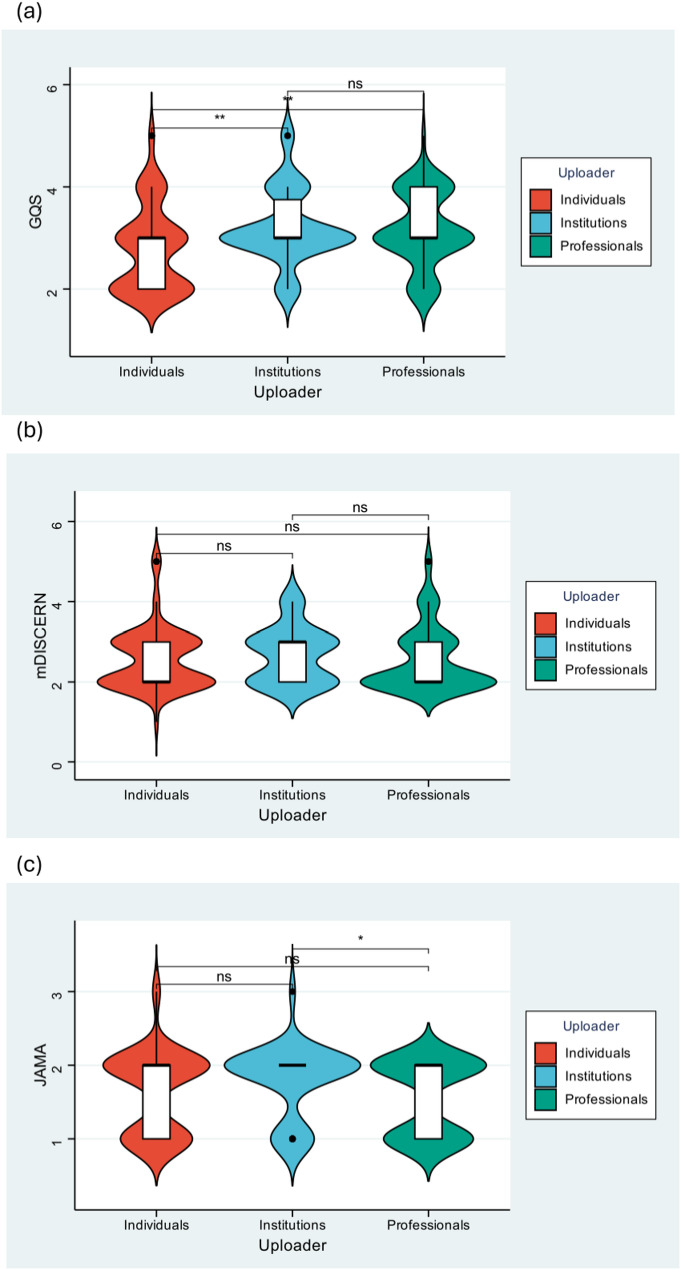
Quality scores and distribution of videos related to NIPT across different uploaders: (**a**) GQS, (**b**) mDISCERN, (**c**) JAMA

Video quality varied with different sources. The median GQS of videos uploaded by professionals was 3.00 (3.00,4.00), which was higher than institutional and individual users. In contrast, VIQI score was highest among institutions, while mDISCERN and JAMA score were non-significant across different uploaders (Table 2; Fig. 4).

### Correlation analysis of video quality and characteristics

**Fig. 5:**
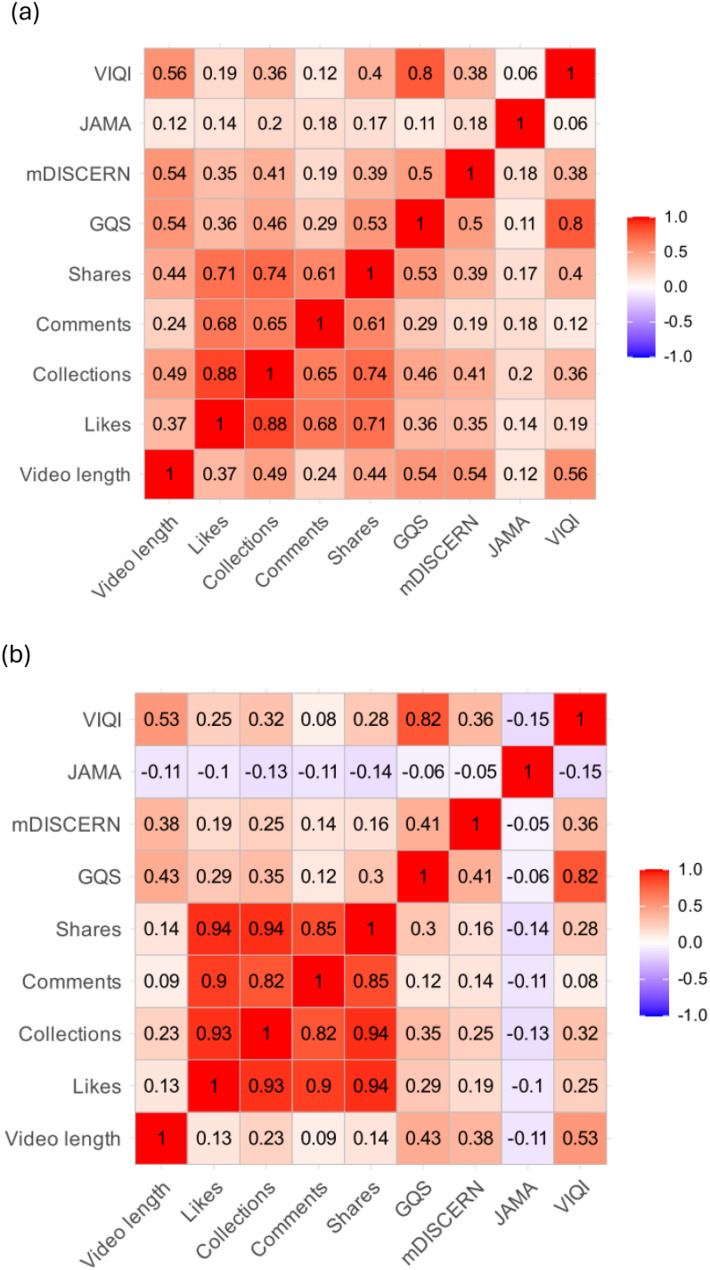
Correlation matrix of video engagement metrics and quality scores on (**a**) Bilibili and (**b**) TikTok

Spearman correlation analysis was conducted to examine the relationships between video duration, engagement metrics, and quality scores (Fig. 5). On Bilibili, social engagement metrics (likes, comments, shares, and collections) demonstrated positive intercorrelations (r ranging from 0.61 to 0.88), suggesting that engagement metrics were strongly correlated. However, correlations between these engagement metrics and video quality scores (GQS, mDISCERN, JAMA, VIQI) were relatively weaker (*r* = 0.12–0.53), indicating that popular videos were not necessarily of high quality. Video duration showed similarly weaker associations with both engagement and quality measures on Bilibili (*r* = 0.12–0.56). In contrast, TikTok exhibited stronger positive correlations among all engagement metrics (*r* = 0.82–0.94), reflecting highly synchronized user interaction patterns on this platform. Despite this strong internal consistency, correlations between engagement metrics and quality scores on TikTok remained weak or inconsistent. Notably, JAMA scores demonstrated negative correlations with all social engagement metrics (r ranging from − 0.14 to -0.10), suggesting that videos with greater transparency received less viewer engagement. Similarly, VIQI scores showed weak positive correlations with engagement (*r* = 0.08–0.32), while GQS and mDISCERN exhibited more positive associations (*r* = 0.12–0.35). These findings collectively reveal a concerning pattern across both platforms: social engagement metrics do not reliably reflect the informational quality and reliability of NIPT-related content, with popular videos often lacking adequate transparency and educational value.

## Discussion

This cross-sectional study provides the first systematic evaluation of the quality and reliability of short videos concerning Non-Invasive Prenatal Testing (NIPT) on two major Chinese social media platforms, TikTok and Bilibili. Our analysis of 180 videos reveals a complex informational landscape, which was characterized by significant inter-platform differences in content sourcing and presentation. Bilibili videos demonstrated significantly longer duration and higher quality scores across multiple assessment instruments (GQS, mDISCERN, VIQI) compared to TikTok content. Conversely, TikTok videos generated substantially higher social engagement metrics, including likes, collections, shares, and comments. This disparity reflects platform-specific user behaviors: Bilibili users had greater acceptance of medium-to-long-form educational content, whereas TikTok’s format primarily aligns with entertainment-focused, fragmented viewing habits for rapid social interaction [7]. Regarding video source, those uploaded by medical professionals not only had higher quality but also gained more social interactions, indicating that professional knowledge background and content credibility are important factors affecting user acceptance and participation behavior [14]. This suggests that active engagement by healthcare providers on short-form video platforms can positively influence the baseline quality of available information. Despite good overall quality, the VIQI scores on both platforms were less than expectation, and the incomplete information to the public may affect viewer’s understanding and decision-making.

A particular concern across both platforms is the weak to negligible correlation between user engagement metrics and video quality and reliability. This finding replicates a pattern reported in studies on tachycardia, and gestational diabetes mellitus [5, 11]. Such a discrepancy indicates that algorithmic promotion and user interaction on social media platforms are largely driven by factors such as emotional appeal, narrative simplicity, or entertainment value, rather than informational accuracy. As a result, emotionally engaging but potentially inaccurate content may receive disproportionate visibility, creating what can be described as a “misinformation ecosystem”: the most widely viewed videos are not necessarily the most reliable. This phenomenon is particularly concerning in the context of prenatal genetic testing. First, the complexity nature of NIPT requires precise explanation, which short-form videos may oversimplify. Second, expectant parents making decisions about NIPT may be psychologically vulnerable and therefore more likely to engage with emotionally appealing content that could increase their anxiety. Third, misunderstanding NIPT results may lead to overtreatment or false reassurance, potentially influencing subsequent clinical decision-making.

This study extends the growing body of literature on social media health information by focusing specifically on prenatal genetic testing, a field where misinformation may have potentially serious consequences for reproductive decision-making. Compared with topics such as endoscopic procedures or some certain disorder, where treatment pathways are relatively established, information related to NIPT may directly influence whether patients pursue invasive diagnostic procedures or make critical pregnancy-related decisions [15, 16]. Therefore, the quality of publicly available information is closely linked to informed consent and patient autonomy in prenatal care. Users should be encouraged to prioritize content from verified healthcare institutions and professionals and to cross-reference information with authoritative sources. For healthcare providers and educators, there is a clear mandate and opportunity to actively participate in these digital spaces. Creating accurate, engaging content is an essential component of patient education. For platform regulators and public health authorities, our results add to the compelling evidence for the need for smarter curation [5, 10, 17, 18]. Potential reforms include implementing prominent verification systems for medical professionals, developing algorithm weights that factor in quality indicators, and creating official partnerships to promote evidence-based content.

This study has several strengths. First, the topic of NIPT is distinct in several ways: it involves genetic testing with potential implications for ongoing pregnancy, requires understanding of statistic concepts (sensitivity, specificity, positive predictive value), and carries unique psychological and ethical considerations. Second, the comparative design between two dominant Chinese platforms with distinct user cultures and algorithmic behaviors moves beyond single platform analyses common in prior literature [10, 19]. China has the world’s largest population of Internet users; a substantial proportion are of reproductive age [20]. Simultaneously, China has also emerged as the leading global market for NIPT [21, 22]. Therefore, examining NIPT-related content on Chinese social media platforms may provide valuable insights for health communication professionals and platform regulators alike. The addition of correlation analysis to descriptional analyses further bridges the engagement-quality gap. Third, we applied four validated assessment tools to provide a multi-dimensional analysis, which can complement each other’s limitations [17].

However, there are a few limitations. Firstly, the cross-sectional design offers only a snapshot of a dynamic environment. The dynamic nature of social media algorithms means results and available videos may change fast. Secondly, the use of a single Chinese search term may omit other relevant content using colloquial synonyms. Furthermore, despite assessed video quality and reliability with various instruments, there can be subjective in evaluation. We tried to reduce the inherent subjectivity by dual-independent reviewing, thorough discussion and calculation of inter-rater coefficients.

## Conclusion

In conclusion, the overall quality and reliability of NIPT-related short videos on TikTok and Bilibili remain suboptimal, despite their high user engagement. Bilibili videos demonstrated relatively better informational quality, whereas TikTok content achieved substantially higher interaction metrics that were not consistently associated with higher quality or reliability. These findings highlight the need for strengthened content regulation, improved platform curation mechanisms, and increased participation of qualified professionals to ensure accurate and trustworthy dissemination of NIPT information to the public.

## Supplementary Information

Supplementary Material 1.

## Data Availability

All data used in this study are publicly available on relevant social media platforms.

## Abbreviations

GQS: Global Quality Score
JAMA: Journal of the American Medical Association
LLM: Large language models
mDISCERN: Modified Decision-making Information Support Criteria for Evaluating the Reliability of Non-randomised Studies
NIPT: Non-Invasive Prenatal Testing
SPSS: Statistical package for social sciences
VIQI: Video Information and Quality Index
