# Supplementary material for: Evaluation of short video quality and reliability of non-invasive prenatal testing on TikTok and Bilibili platforms: a cross-sectional study
Source: BMC Pregnancy Childbirth. 2026 Mar 20;26:468. doi: 10.1186/s12884-026-08965-x (PMC13126714; doi:10.1186/s12884-026-08965-x)
Supplement: Supplementary file 1 — Supplementary Material 1. [file 12884_2026_8965_MOESM1_ESM.docx]

**Supplementary Table 1.** The inter-rater reliability of video quality scores

|  | **GQS** | **mDISCERN** | **JAMA** | **VIQI** |
| --- | --- | --- | --- | --- |
| **Cohen’s k coefficients** | 0.675 | 0.703 | 0.763 | 0.627 |

GQS: Global Quality Scale, Mdiscern: Modified Decision-making Information Support Criteria for Evaluating the Reliability of Non-randomised Studies, JAMA: Journal of the American Medical Association, VIQI: Video Information and Quality Index.
